# Threatened Death from Chloroform

**Published:** 1869-11

**Authors:** 


Threatened Death from Chloroform.—The *British Medical Journal*, of Sept. 11, says: Recently, at King's College Hospital, London, there was a very narrow escape from death by chloroform. The patient was a healthy man, aged 30, who was to undergo the operation of removal of a tumor from the front of the leg, by Mr. Henry Smith. As the inhalation proceeded, the patient began to struggle so violently that it required the assistance of several dressers to prevent him from throwing himself from the table. He, however, became insensible to pain; and Mr. Smith proceeded with the dissection, but was compelled to desist, in consequence of the violent movements of the patient. The chloroform was now entirely suspended; but, notwithstanding this, the man's face became suddenly livid, then changed to a deep purple color, respiration and pulse completely stopped, and death had apparently taken place. Mr. Smith at once thrust his finger to the top of the windpipe, got forward the tongue, and assistants commenced artificial respiration by the movements recommended by Dr. Silvester. The naked chest was vigorously flipped with a wet towel. For a brief period these measures seemed to produce no effect; but after a short time there was a slight improvement in the complexion, when the efforts were redoubled, and all were delighted to find the apparently dead man slowly respiring. In two or three minutes more, the man had so far recovered that Mr. Smith was able to complete the operation, although, of course, no chloroform was exhibited. In some remarks after the operation, Mr. Smith referred to the narrow escape of the patient, and said it illustrated the danger which will occasionally attend chloroform, however carefully given, more especially in those cases where its exhibition is followed by a great amount of struggling. It was necessary to be particularly careful with it when this occurred; he had seen other narrow escapes exactly under the same circumstances.

